# *In Vivo* Evolution of Tumor-Derived Endothelial Cells

**DOI:** 10.1371/journal.pone.0037138

**Published:** 2012-05-18

**Authors:** Terence F. McGuire, Gangadharan B. Sajithlal, Jie Lu, Robert D. Nicholls, Edward V. Prochownik

**Affiliations:** 1 Division of Hematology/Oncology, Department of Pediatrics, Children’s Hospital of Pittsburgh of UPMC, Pittsburgh, Pennsylvania, United States of America; 2 Birth Defect Laboratories, Division of Genetics, Department of Pediatrics, Children’s Hospital of Pittsburgh of UPMC, Pittsburgh, Pennsylvania, United States of America; 3 Department of Human Genetics, Graduate School of Public Health, University of Pittsburgh, Pittsburgh, Pennsylvania, United States of America; 4 Department of Microbiology and Molecular Genetics, The University of Pittsburgh School of Medicine, Pittsburgh, Pennsylvania, United States of America; 5 The University of Pittsburgh Cancer Institute, Pittsburgh, Pennsylvania, United States of America; University Magna Graecia, Italy

## Abstract

The growth of a malignant tumor beyond a certain, limited size requires that it first develop an independent blood supply. In addition to providing metabolic support, this neovasculature also allows tumor cells to access the systemic circulation, thus facilitating metastatic dissemination. The neovasculature may originate either from normal blood vessels in close physical proximity to the tumor and/or from the recruitment of bone marrow-derived endothelial cell (EC) precursors. Recent studies have shown that human tumor vasculature ECs may also arise directly from tumor cells themselves and that the two populations have highly similar or identical karyotypes. We now show that, during the course of serial *in vivo* passage, these tumor-derived ECs (TDECs) progressively acquire more pronounced EC-like properties. These include higher-level expression of EC-specific genes and proteins, a greater capacity for EC-like behavior *in vitro*, and a markedly enhanced propensity to incorporate into the tumor vasculature. In addition, both vessel density and size are significantly increased in neoplasms derived from mixtures of tumor cells and serially passaged TDECs. A comparison of early- and late-passage TDECs using whole-genome single nucleotide polymorphism profiling showed the latter cells to have apparently evolved by a process of clonal expansion of a population with a distinct pattern of interstitial chromosomal gains and losses affecting a relatively small number of genes. The majority of these have established roles in vascular development, tumor suppression or epithelial-mesenchymal transition. These studies provide direct evidence that TDECs have a strong evolutionary capacity as a result of their inherent genomic instability. Consequently such cells might be capable of escaping anti-angiogenic cancer therapies by generating resistant populations.

## Introduction

The growth of a tumor beyond a certain small size is highly dependent upon the development of an independent vasculature [1]–[4]. This “angiogenic switch”, which may not be activated until years after the initial neoplasm has developed [5], [6], has until recently generally been viewed as having two origins. The first, termed “sprouting” angiogenesis, or simply angiogenesis, is a consequence of the re-directing of pre-existing neighboring blood vessels into the tumor bed, whereas the second, termed vasculogenesis, stems from the recruitment of bone marrow-derived endothelial cell (EC) precursors [7], [8]. Both processes may operate independently and concurrently and are regulated by specific inductive signals originating from the tumor and/or its associated inflammatory cell infiltrate [8], [9]. Once developed, the neo-vasculature becomes the tumor’s main source of oxygen and nutrients, whose delivery is no longer diffusion-dependent. It also serves as a major portal for tumor cell invasion of the systemic circulation, thus abetting metastasis [3], [6].

Given the neovasculature’s importance in supporting many of the tumor’s most critical metabolic needs and functions, much effort has been devoted to the design of anti-angiogenesis therapies [10], [11]. One attraction of this approach is that the cells comprising the vasculature are presumed to be genomically stable and thus less predisposed to the development of the therapy resistance commonly encountered in tumor cells [2], [3]. Despite this major theoretical advantage, clinical trials with these approaches have extended patient survival only modestly and the eventual development of resistance is actually quite common, although its cause remains largely unexplored [12]–[15].

We and others have described a third origin for the EC population of several tumor types in the form of “tumor-derived ECs” (TDECs) [16]–[18], which arise stochastically from tumor epitlelium. TDECs express EC-specific markers, contain Weibel-Palade bodies, form tube-like structures in semi-solid media, and incorporate into functional tumor vasculature. These properties distinguish TDECs from tumor cells undergoing vasculogenic mimicry, a reversible process involving a partial epithelial-mesenchymal transition (EMT) [19], [20]. As expected for actual tumor cell descendants, TDEC karyotypes are distinctly abnormal and contain tumor-specific chromosomal markers. These findings suggest that, like their ancestral tumor cells, TDECs might possess inherent genomic instability (GI) and thus be prone to develop resistance to anti-angiogenic therapies [16]–[18]. Consistent with this idea, we show here that upon continued in vivo serial passage, the TDEC population acquires a more pronounced EC-like phenotype. Whole genome single nucleotide polymorphism (SNP) array analysis indicates that serially-passaged TDECs also acquire a small number of genomic amplifications and deletions. The known genes contained within these affected regions are disproportionately involved in functions pertaining to tumor suppression, vasculogenesis, and EMT. These findings support the idea that the long-term propagation of TDECs leads to the emergence of a more functionally robust clonal population that is facilitated by underlying GI.

## Results

### TDEC Recovery Increases with Serial Passage

**Figure 1:**
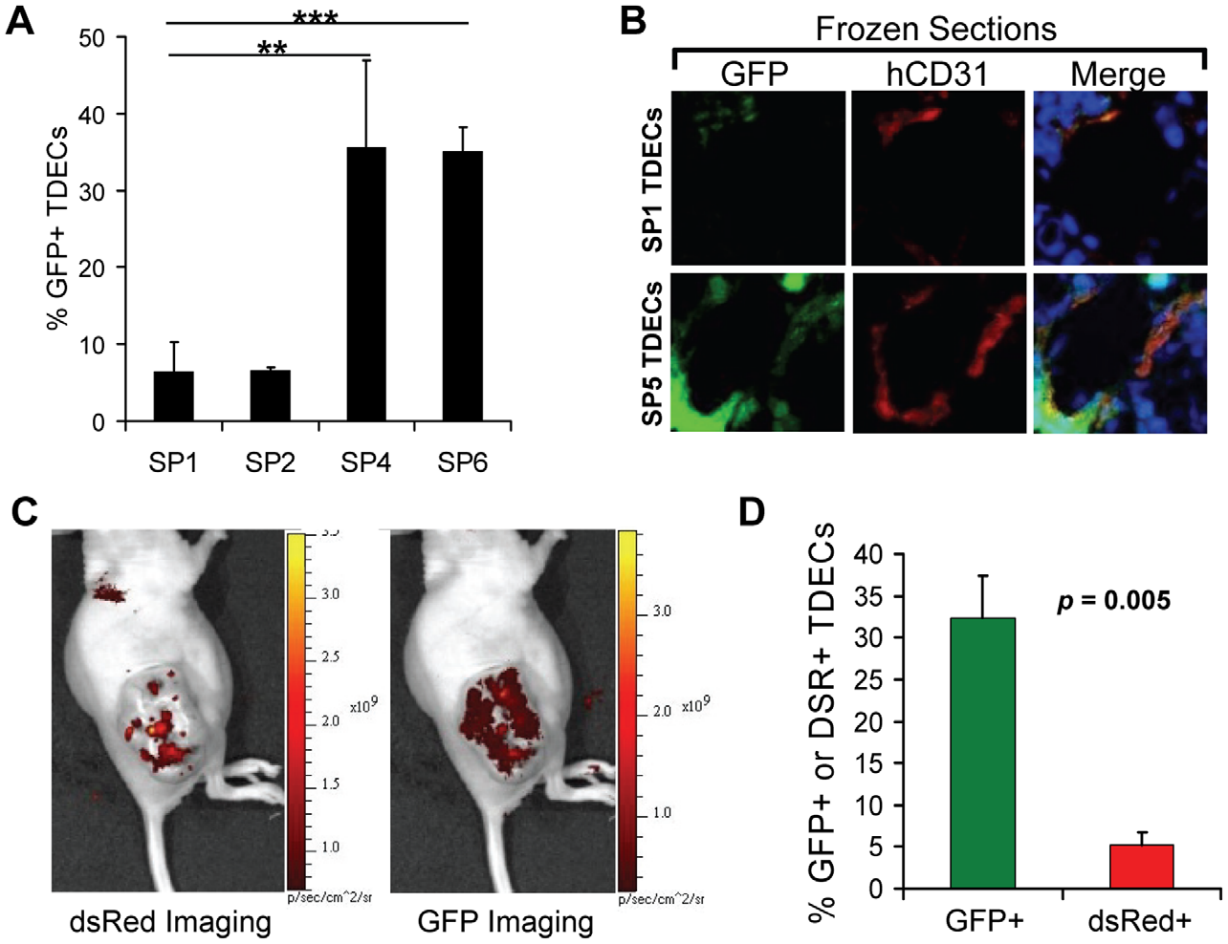
Increased tumor re-populating ability of serially-passaged TDECs. (A) Human and murine TAECs were isolated from initial H460 lung cancer xenografts by CD31 immuno-affinity purification [16]. The latter were then eliminated by propagating the cells in G-418 and the resultant GFP+, G418-resistant human TDECs were then serially passaged *in vivo* with a 20-fold excess of non-GFP-tagged H460 tumor cells. At the indicated passage numbers, the percent of GFP+ TDECs was determined 1–2 days after isolation and before the addition of G-418. The results shown represent the average values obtained from 3–5 fields (±SEM) with a total of 738, 761, 128, and 296 cells counted for SP1, SP2, SP4, and SP6, respectively. The statistical analysis was performed using a two-tailed Student’s *t* test (**, *p*<0.01; ***, *p*<0.0001). (B) Photomicrographs of typical blood vessels from H460 tumor xenografts established as described in *A* for serial passages SP1 and SP5. Frozen sections were visualized for GFP and stained for human-specific CD31 (hCD31) (red) and DAPI (blue). Images were taken at 20× magnification. (C) Live animal imaging of tumor xenografts arising in a representative mouse co-inoculated with an equal number of dsRed-tagged SP0 TDECs and GFP-tagged SP6 TDECs plus a 20-fold excess of untagged H460 tumor cells. (D) Quantification of the percent red SP1 versus the percent green SP7 TDECs isolated from three tumors. The graph represents the mean values (±SEM) with the *p* value determined using a one-tailed Student’s *t* test.

The H460 human lung cancer cell line was originally tagged with lentiviral vectors separately encoding green fluorescent protein (GFP) and resistance to G418 [16]. Tumor xenografts from these cells were initially grown in immuno-compromised nude mice and the total human and murine EC populations were isolated with anti-CD31-coupled magnetic beads as previously described [16]. Approximately 40% of the total “tumor-associated ECs” (TAECs) expressed GFP, were G418-resistant and contained only human chromosomes, thus indicating that they were true TDECs [16]. These “serial passage 0” (SP0) TDECs were then mixed with a 20-fold excess of untagged H460 cells and re-propagated with tumor xenografts. Upon reaching maximal allowable size, each tumor was excised and its its total TAEC population, now referred to as SP1, was again isolated. The GFP+ TDECs, which comprised approximately 5% of the entire TAEC population, were then purified and expanded for several days in G418-containing medium, combined again with a 20-fold excess of untagged H460 tumor cells and propagated as tumor xenografts. Several such serial *in vivo* passages were performed with the percent of retrieved GFP+ TDECs in the total TAEC population being determined periodically. While the earliest passage TDECs (SP1 and SP2) consistently yielded about 5% GFP+ TDEC recovery, later passages (SP4–SP6) provided for recovery rates in excess of 30% (Figure 1A). This was consistent with the observation that these later passage TDECs were more frequently associated with tumor blood vessel endothelium (Figure 1B).

To establish further that the late passage cells could out-compete their early passage counterparts, we derived SP0 TDECs from an H460 tumor tagged with a red fluorescent protein (dsRed) rather than GFP. The SP0 (dsRed+) TDECs and SP6 (GFP+) TDECs were then mixed in equal numbers, combined with untagged H460 cells at a 1∶20 ratio as before, and grown as tumor xenografts. Just prior to total TAEC harvesting, fluorescence imaging of the mouse was performed using a Xenogen IVIS-200 *in vivo* imaging system. As seen in Figure 1C, the dsRed signal localized to discrete regions, that recapitulated the distribution previously seen for tumors containing early passage TDECs [16], while the GFP signal was more evenly distributed throughout the tumor. Following the isolation of TAECs, the red and green TDEC populations were quantified by direct UV microscopy. In three independent tumor xenografts, there was an average of >6-fold greater abundance of GFP+ TDECs than of dsRed+ TDECs (Figure 1D). Taken together, these observations indicate that late passage TDECs outcompete early passage TDECs, even when the two populations co-exist under identical *in vivo* conditions.

### Late Passage TDECs Express Higher Levels of some EC-specific Markers

**Figure 2:**
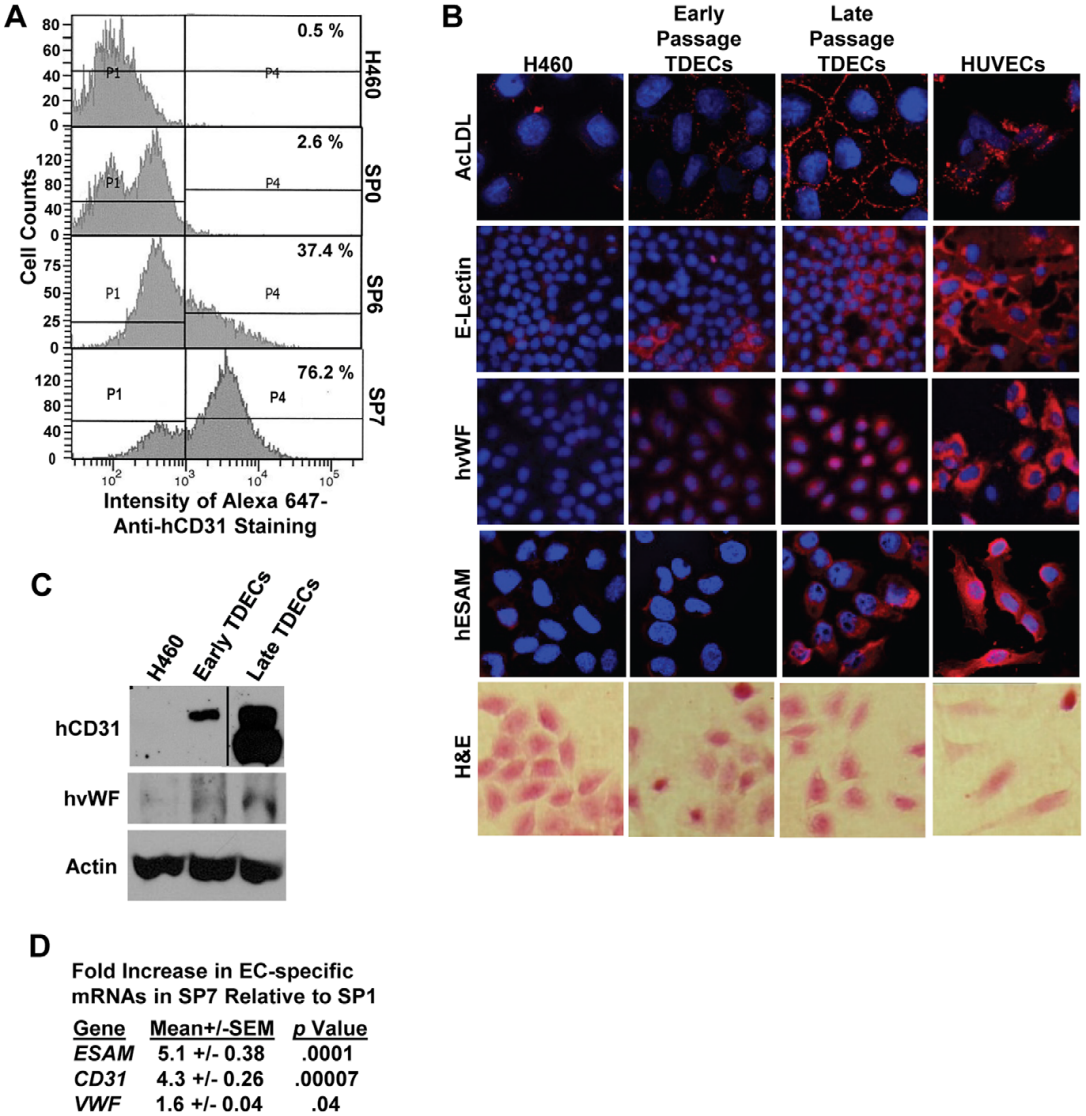
EC-specific marker expression increases in serially-passaged H460-derived TDECs. (A) Cell surface staining for hCD31. TDECs from the indicated serial passages were isolated and the cell surface expression of the EC-specific marker hCD31 was then assessed by flow cytometry. (B) AcLDL uptake, E-lectin binding and anti-human vWF (hvWF) and anti-human ESAM (hESAM) immuno-staining were assessed in early and late TDECs using confocal microscopy (16). HUVECs were included as positive controls. Nuclei were stained with DAPI (blue). H&E staining under light microscopy was used to compare the morphologies of H460 tumor cells, TDECs, and HUVECs. Images were obtained at 20−40× magnification. (C) Expression of CD31 and vWF assessed by immuno-blotting. Early- and late-passage TDECs were examined by immuno-blotting for hCD31 and hvWF, as previously described [16]. All lanes in the hCD31 blot were from the same X-ray film. β-actin levels were measured on the same samples and served as a loading control. (D) qRT-PCR was used to assess mRNA expression for the EC markers ESAM, CD31, and VWF in late passage TDECs relative to early passage TDECs. Values shown represent the average of at least triplicate reactions and *p* values were from a standard Student’s *t* test.

We previously reported that early passage H460-derived TDECs, as well as those originating from several other tumor cell types, stably express numerous EC-specific markers [16]. Having demonstrated that late passage TDECs outcompete early passage TDECs and incorporate into the tumor vasculature more efficiently, we next asked whether they do so in association with higher level expression of these markers. Thus, we used flow cytometry to follow the cell surface expression of CD31 on TDECs during the course of serial *in vivo* passage and observed that it increased by nearly 30-fold (Fig. 2A). This increase was confirmed by immunoblotting (Fig. 2C). Similarly, several other EC-specific markers, including uptake of acetylated LDL (AcLDL), expression of E-lectin receptor, von Willebrand’s factor (vWF) and EC-selective adhesion molecule (ESAM), also increased over the course of serial passage, rivaling the levels observed in human umbilical vein endothelial cells (HUVECs) and coinciding with the acquisition of a more EC-like morphology (Fig. 2B). Finally, an examination of EC-specific transcripts, which had previously shown 2 to100-fold increases in SP0 TDECs relative to parental H460 tumor cells [16], showed additional 1.6 to 5.1-fold increases in expression between SP1 and SP7 TDECs (Figure 2D). Taken together, these studies demonstrate further up-regulation of numerous EC specific markers during the course of TDEC serial passage.

### Late Passage TDECs form more Complete Tube-like Structures and Become Hypoxia-responsive

**Figure 3:**
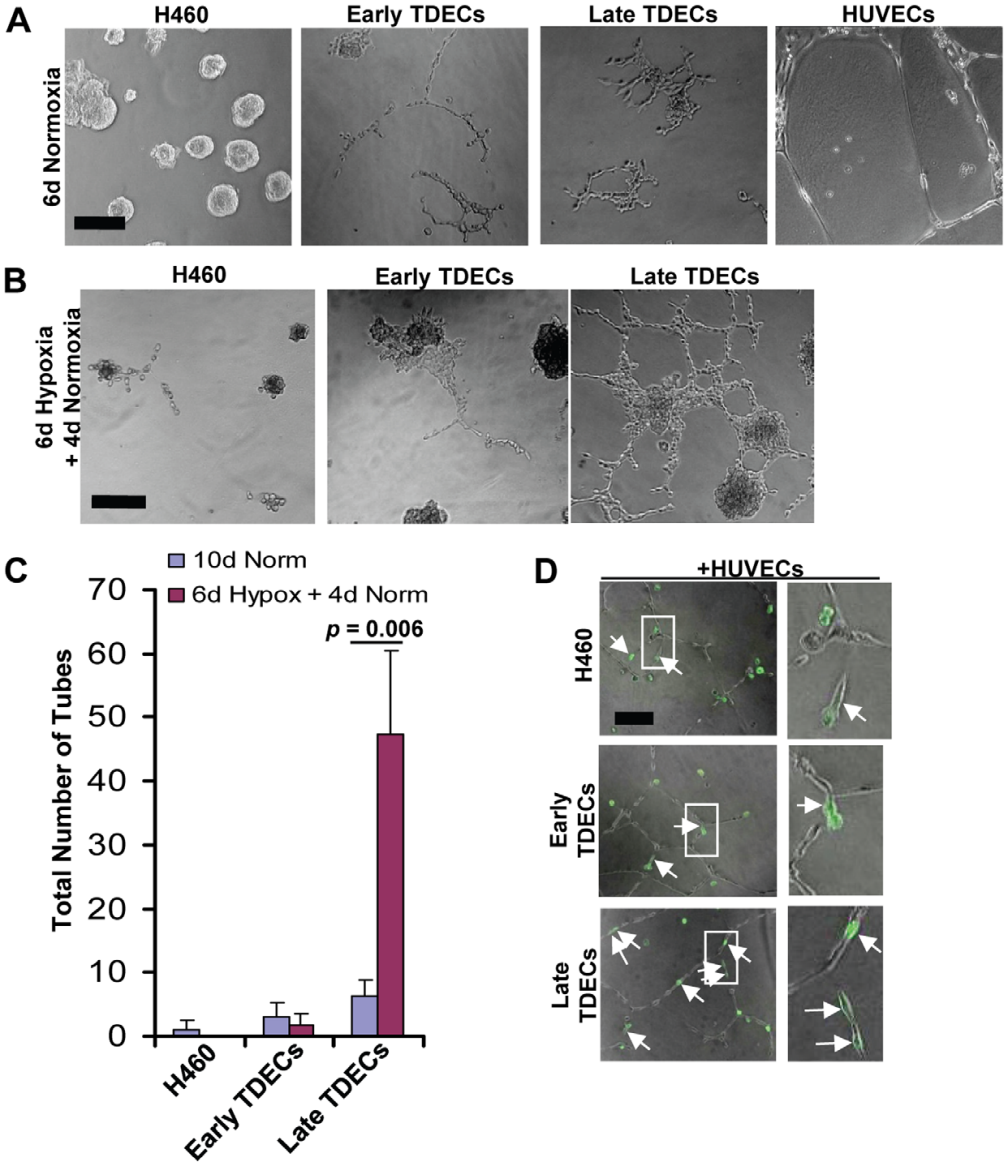
Serially-passaged TDECs demonstrate increased tube formation. (A) H460 tumor cells, early and late passage TDECs, and HUVECs were plated in Matrigel as previously described [16] and incubated under standard normoxic conditions for six days. Typical light microscopic fields are shown. All photos are shown at identical magnifications with the black bar representing 100 µm. (B) The experiment from *A* was repeated except that cultures were incubated for six days under moderately hypoxic conditions (1% O_2_) followed by an additional four day recovery period under normoxic conditions. (C) The total number of tubes from *B* as well as those from parallel plates of cells cultured 10 d in normoxia (control) were quantified. The plot depicts the mean values (±SEM) from three independent experiments. The *p* value was determined for only the late TDECs using a one-tailed Student’s *t* test. (D) GFP-tagged H460 tumor cells or early or late TDECs (1,000 cells) incubated alone or mixed with non-GFP-tagged HUVECs (10,000 cells). The cells were then cultured for two days under normoxic conditions in a standard Matrigel-based tube assay. Brightfield and UV fluorescence photographs were taken and the merged images shown. In the first two cases, the *white arrows* indicate GFP+ tumor cells or early-passage TDECs that lie adjacent to but have not incorporated into small groups of HUVECs. *Arrows* in the bottom two panels indicate late passage TDECs that have incorporated into HUVEC tubes. Note that when GFP-tagged H460 tumor cells and early and late GFP+ TDECs were plated without HUVECs, they remained rounded after 2 days of culture on Matrigel (see Figure S1). All photos are shown at identical magnifications with the black bar representing 100 µm. Enlargements of representative views (areas in *white rectangles*) are also shown. Original images in *panels A, B,* and *D* were taken at 10× magnification.

One hallmark of ECs is their ability to form 3-dimensional tube-like structures resembling capillaries in semisolid media [21], [22]. When cultured under standard normoxic conditions for six days in Matrigel, H460 tumor cells formed only acini, while both early and late passage H460-derived TDECs generated abortive, network-like structures only partially resembling those formed by HUVECs (Figure 3A and reference 16). In contrast, when TDECs were cultured for 6 days under moderately hypoxic conditions (1% O_2_) followed by a 4 day normoxic recovery period, a more complete HUVEC-like tube pattern was observed with late, but not early, passage TDECs (Figure 3B and C). TDEC serial passage thus is associated with an enhanced tube-forming ability, which requires hypoxic stress for optimal expression.

To explore further the *in vitro* EC-like properties of TDECs, we co-cultured HUVECs with GFP-tagged H460 cells or with early or late passage H460-derived GFP+ TDECs. We reasoned that, during propagation in Matrigel, HUVECs might provide supportive factors that would enhance the ability of TDECs to form tube-like structures. After 2 d of isolated culture, H460 and early and late passage TDECs cultured alone remained predominantly single, rounded cells and had not formed tubes (Figure S1). However, when co-cultured with HUVECs, we observed a greater tendency of late passage TDECs to elongate and incorporate into (not just associate with) HUVEC tubes compared to either early passage TDECs or H460 cells (Figure 3D). These results are consistent with those shown in Figure 3A–C and indicate that late passage TDECs are more responsive to and cooperate better with neighboring HUVECs than early passage TDECs.

### Enhanced Vascularization Potential of Serially-passaged TDECs

**Figure 4:**
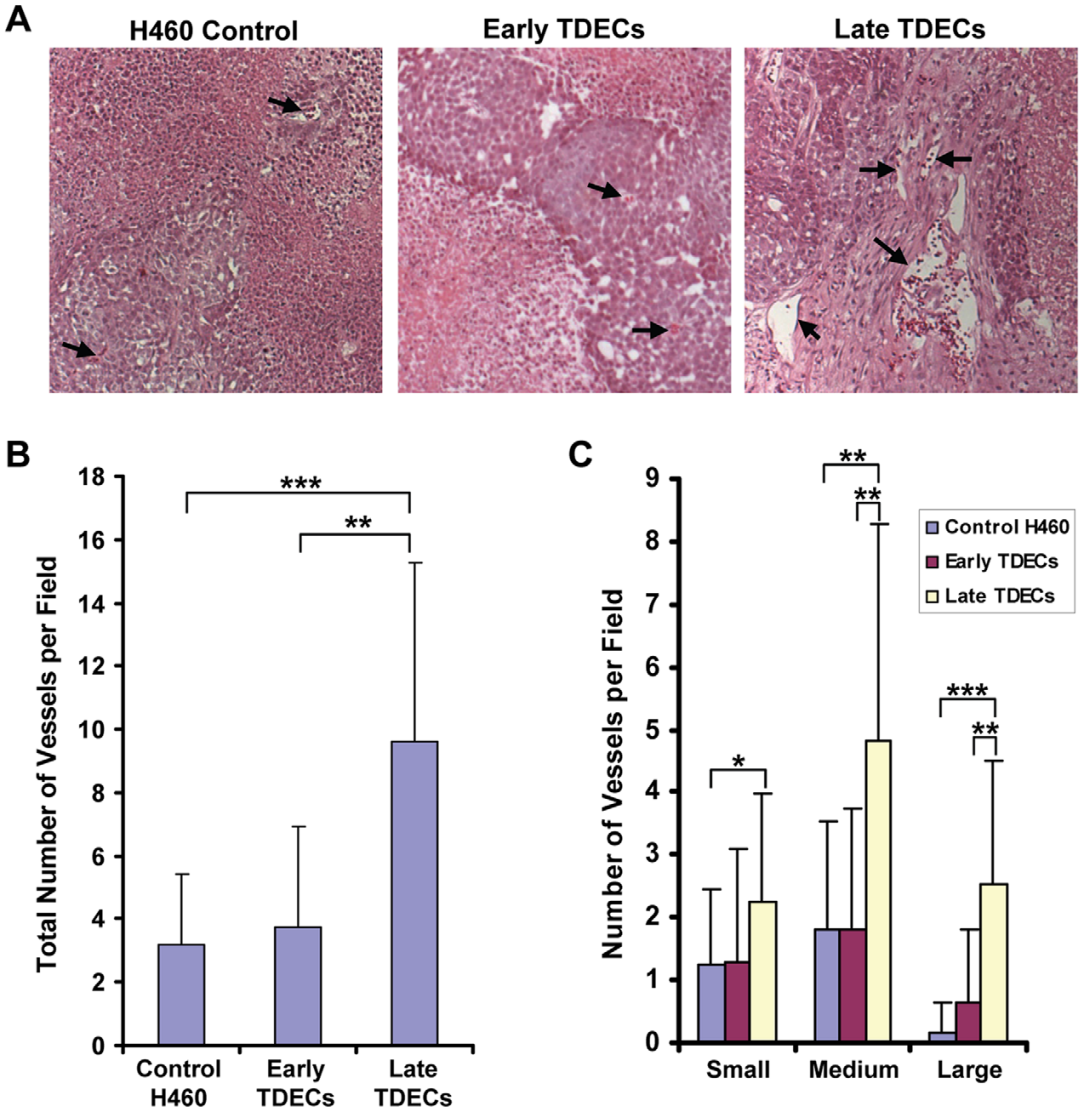
Tumor xenograft blood vessel density and size increase with continued serial passage of TDECs. Tumor xenografts were established from inoculating a mix of non-GFP-tagged H460 cells (95%) and early- or late-passage TDECs (5%) into nude mice. Control tumor xenografts were similarly derived from inoculating non-GFP-tagged H460 cells and 5% GFP+ H460 cells. Paraffin-embedded sections were then prepared from the xenografts and stained with hematoxylin and eosin (A) so that tumor blood vessels could be easily identified. Typical examples of blood vessels from each xenograft type are indicated by *arrows.* The total number of tumor blood vessels (B), as well as the number of vessels that were small (≤25 µm), medium (25–100 µm), and large (≥100 µm) as measured by their longest dimension (C), were quantified from between 19 and 25 individual fields for each tumor type. The graphs represent the mean number of vessels per field (±SEM) and the statistical analysis was performed using a two-tailed Student’s *t* test (*, *p*<0.05; **, *p*<0.001; ***, *p*<0.0001).

We next examined whether the tumors arising from H460 tumor cells containing a 5% contribution of late-passage TDECs might be more highly vascularized than those arising from a similar mix with either early-passage TDECs or GFP-tagged tumor cells (control tumors). Paraffin-embedded sections of tumor xenografts were stained with hematoxylin and eosin to identify tumor blood vessels. We found that tumors originating from the inocula containing late-passage TDECs tended to be supplied by blood vessels that were both denser and larger than those seen in the other two tumor groups (Figure 4A–C). Thus, continued serial passage of TDECs is associated with an enhanced ability to establish a functional tumor vasculature.

### Late Passage TDECs have Defined Genomic Gains/deletions that Affect Gene Expression

**Figure 5:**
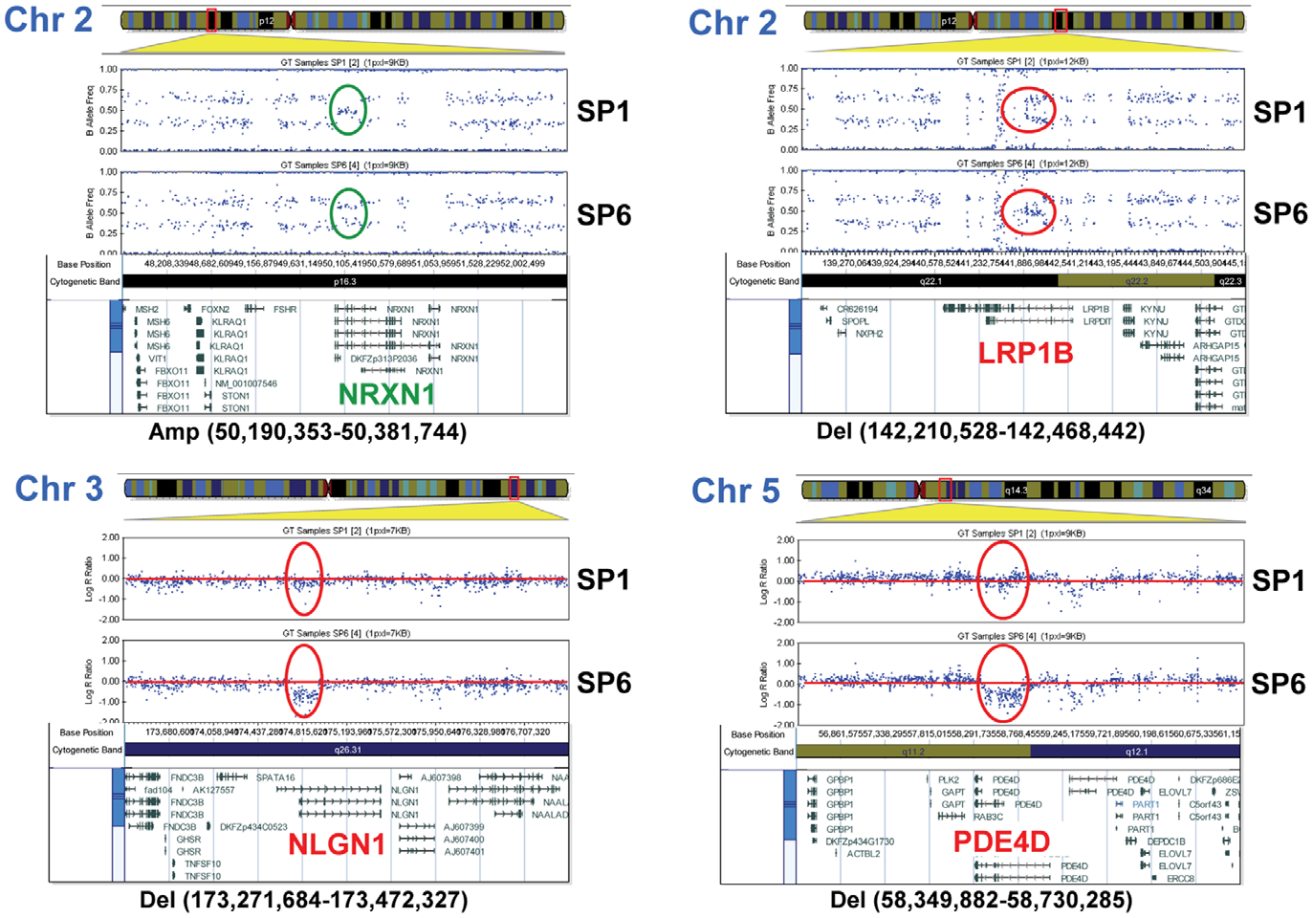
Analysis of SNP array profiles for four representative genomic alterations. Shown are genomic alterations found in late passage TDECs on chromosomes 2, 3, and 5. In each case, the results shown (either Log R Ratio or B Allele Frequency) represent those that most clearly depict the chromosomal alteration, although both types of analyses were used for interpretations. SNP array profiles for all genomic alterations detected are shown in Figures S2, S3, S4, S5, S6, and S7. Chromosomal gains (green) and deletions (red) in late-passage TDECs relative to early passage TDECs are indicated as well as the base positions of the alterations on the chromosomes. The expanded regions below the SNP array plots show the genes within and adjacent to the sites of chromosomal alteration. The boundaries of the chromosomal alterations were determined using GenomeStudio software (version v2009.2) to identify SNP coordinates and the Ensembl Genome Browser 57 database.

As late passage TDECs have more pronounced EC-like phenotypes than their early passage counterparts, it seemed possible that serial propagation might be selecting for a subpopulation of cells inherently better at contributing to tumor blood vessel formation. This process could be accelerated if TDECs, like the tumor cells from which they originate, were more genomically unstable than host-derived ECs. To test this hypothesis, we compared genome-wide SNP profiles of H460 cells and early and late passage TDECs. No differences were observed in the SNP profiles of early-passage TDECs (SP0 and SP1) and H460 cells, whereas late-passage TDECs (SP5 and SP6) showed identical genomic alterations (Figures S2, S3, S4, S5, S6, and S7). Aside from the loss of one copy of chromosome 14, these consisted of a relatively small number of deletions and gains (8 and 2, respectively) ranging in size from approximately 56 kb to 435 kb (Figure 5, Table 1, and Figures S2, S3, S4, S5, S6, S7). Seven of the ten genomic gains/deletions in late-passage TDECs were found to be intragenic (Table 1), whereas nine additional genes were located within the immediate vicinity of the affected loci (Table 1). Interestingly, five of the genes with intragenic alterations and 6 of the adjacent genes have been previously linked to tumorigenesis, vasculogenesis or the EMT and several have also been implicated in neuronal development (Table 1) [23]–[38]. Taken together, these results indicate that late passage TDECs appear to arise through a process of clonal selection and possess discrete regions of genomic gain/deletion commonly involving genes with functions potentially relevant to the transition from tumor epithelium to ECs.

**Table 1:** Summary of genomic alterations in late passage H460 TDECs.

Chromosomal Position &Alteration	Gain/Del	Possible Genes Affected	GenBank Accession Number	Location of Alteration Relative to Altered Gene	Proposed Biological Role(s)	Refs.
						
2p16.3 (50,190,353-50,381,744)	Gain	*NRXN1*	AB011150	Exons 20-22	nervous and vascular systems	*23*
2q22.1 (142,210,528-142,468,442)	Del	*LRP1B*	NM_018557	Exon 3	deleted in cancers	*24*
3q13.31/32 (117,059,636-117,473,977)	Del	*LSAMP*	NM_002338	5′	tumor suppressor, VEGFR1 regulator	*25*
		*LSAMP-AS3*		5′	noncoding LSAMP antisense RNA	*25*
		*LSAMP-AS4*		5′	noncoding LSAMP antisense RNA	*25*
3q26.31 (173,271,684-173,472,327)	Del	*NLGN1*	NM_014932	Exons 1b-2	nervous and vascular systems	*23*
4q34.3 (182,009,132-182,158,533)	Del	*LINC00290*		Intergenic		
5q11.2 (58,349,882-58,730,285)	Del	*PDE4D*	NM_001104631	Exons 2–5	promotes survival, proliferation, EMT	*26*
		*RAB3C*	NM_138453	3′	exocytosis regulation	*27*
		*PART1*	NR_024617	5′	(long non-coding RNA)	
		*DEPDC1B*	NM_018369	5′	deleted in sporadic breast cancer	*28*
		*ELOVL7*	NM_024930	5′	LCFA elongase, prostate cancer	*29*
		*ERCC8*	NM_000082	5′	DNA repair	*30*
5q34 (164,826,163-165,260,961)	Del	*ODZ2*	AB032953	5′	a transmembrane protein predominantly expressed in nervous system; deregulatedin lymphoma	*31,32*
9p21.1 (28,378,229-28,587,832)	Del	*LINGO2*	AL353746	Exon 2	transmembrane protein, typicallyexpressed early in development	*33*
13q33.3 (107,685,848-107,885,801)	Gain	*FAM155A*	L10374	Exons 2–3	unknown	
		*ARGLU1*	BC071587	3′	required for estrogen-dependentgene transcription and breast cancer cell growth	*34*
		*EFNB2*	NM_004093	3′	angiogenesis	*35*
		*LIG4*	NM_002312	5′	DNA repair	*36*
14q (entire chromosome)	Loss	All	-	-		
20p12.1 (14,858,910-14,914,578)	Del	*MACROD2*	NM_080676	Intron 5	An O-acetyl-ADP-ribose deacetylase;deleted in colorectal cancer	*37,38*

The nucleotide coordinates of genomic alterations in SP5 and SP6 TDECs were determined by the first and last SNPs that differed from those of early passage TDECs and the precise locations were obtained using the Ensembl Genome Browser 57 database.

Our SNP findings suggested that the expression of genes contained within or adjacent to the amplified/deleted regions of late passage TDECs might differ from that of early passage TDECs and/or parental H460 cells. To investigate this, we performed quantitative real-time polymerase chain reaction (qRT-PCR) on 16 of these transcripts (primer sets are listed in Table S1). Relative to H460 cells, whose expression levels for all transcripts were arbitrarily set at one, we found changes of >1.5-fold in 7 transcripts from early TDECs (*LSAMP*, *DEPDC1B*, *ODZ2*, *FAM155A*, *EFNB2*, *LIG4*, and *MACROD2*) and 6 transcripts from late TDECs (*LRP1B*, *LSAMP*, *NLGN1*, *RAB3C*, *FAM155A*, and *MACROD2*) (Figure S8). Relative to early-passage TDECs, late-passage TDECs showed statistically significant altered expression with 5 of 16 transcripts, namely those of *LRP1B*, *RAB3C*, *FAM155A*, *EFNB2*, and *MACROD2* (Figure S8). The nature of these changes was complex and could not always be predicted by the type of alteration of the respective genomic loci. The most dramatic example was seen in the case of *LRP1B* transcript expression, which was increased 16-fold in late-passage TDECs, despite being associated with a hemizygous allelic loss (Table 1 and Figures S2B and S8).

## Discussion

Although the tumor vasculature has been classically viewed as arising from extra-tumoral sources, numerous studies have demonstrated that some of the cells comprising tumor blood vessels or its stroma display evidence of GI [39]–[45]. Our recent finding that tumor cells can directly transdifferentiate into ECs *in vivo*, and that they stably retain this phenotype, provides one potential explanation for these findings as does the observation that some tumor cells may acquire EC-like properties under particular *in vitro* conditions [16], [46]. These findings are supported by recent observations that a substantial proportion of TAECs from primary human glioblastomas appear to be of tumor cell or tumor stem cell origin [17], [18]. Taken together, these observations suggest that tumor cells may engage in EMT, a potentially intermediate stage in TDEC differentiation. During the EMT, tumor cells lose junctional attachments, become more spindle-like, and acquire more invasive and migratory behaviors [47]–[49]. The tumor cell derivation of TDECs thus provides reason to suspect that they may share the attribute of GI. The combination of functional plasticity and GI could provide the means for TDECs to adapt to microenvironmental challenges imposed by the growing tumor and to evade anti-angiogenic therapies despite being initially sensitive [17].

Based upon previously published [16] as well as our current work, H460 tumor cells and early-passage TDECs are polyclonal populations with essentially no identifiable differences based on karyotypes or SNP profiling. In contrast, late passage TDECs comprise a monoclonal or oligoclonal population with an enhanced revascularization potential. This advantage is only seen upon serial *in vivo* passage as we have found that comparable periods of *in vitro* propagation neither increases the growth rate of early passage TDECs nor allows for clonal populations to emerge (unpublished data) These findings provide further evidence that the *in vivo* micro-environment plays a key role in selecting for TDEC populations that are optimized for participating in tumor neovasculature development.

Late-passage, serially-passaged TDECs possess a limited number of well-defined chromosomal gains and deletions. That the majority of these alterations affect genes that have been implicated in EMT, vasculogenesis, or tumor suppression provides support to a model for which one or more of these genes play a role(s) in the TDEC developmental program. Future studies will be needed to determine the relative contribution of these genes to the TDEC differentiation process.

In addition to the vascular and tumorigenic roles mentioned above, several of the genes affected by TDEC gains/deletions are also active in neurogenesis (see Table 1). This may relate to the tendency of blood vessels and nerves to develop coordinately along similar anatomic pathways and to the fact that nerve-derived signals may also influence vascular patterning [50]–[53]. Moreover, certain highly motile and invasive TDECs, which might be expected to possess a functional advantage for the type of tumor repopulating activity seen in tumor xenografts, have properties that are highly reminiscent of the cells comprising axonal growth cones [50]–[54]. The precise role of these genes in TDEC formation and function and their relationship to the EC and neuronal developmental process remain to be determined.

Although the EC-like properties and behaviors of TDECs clearly increase with *in vivo* passage number (Figures 2 and 3), the cells remain morphologically distinguishable from primary ECs such as HUVECs (Figure 3A and B). The less than complete EC-like appearance of TDECs may reflect the different pathways that drive differentiation of these cells compared to true ECs. Certain key elements of the normal EC differentiation program that are lost or rendered dysfunctional as a consequence of the transformation process and/or the ensuing GI may play independent roles as well. Despite these less than complete phenotypes, the fact that late-passage TDECs are capable of better vascularizing tumor xenografts (Fig. 4) attests to their increasing ability to function within an *in vivo* context.

Together with previous observations [16], the approaches described here should allow a number of interesting questions pertaining to TDEC development and adaptive selection to be addressed. Among these is whether TDECs of diverse tumor types share similar regions of gains/deletions and whether, within these regions, the genes most responsible for TDEC evolution can be better defined with respect to function. The short time-frame over which TDEC evolution occurs should also allow investigation into whether the development of clinical refractoriness to anti-angiogenesis therapies is associated with the emergence of TDEC clonal subsets related to but distinct from those described here.

## Materials and Methods

### Ethics

All mouse studies were conducted according to Animal Welfare Act and the Public Health Service Policy and approved by the University of Pittsburgh’s Institutional Animal Care and Use Committee (IACUC) (Permit Number: 0812276). The animals were housed in pathogen-free units at Children’s Hospital of Pittsburgh Rangos Research Building in compliance with IACUC regulations. Age and gender matched Nu/Nu mice were purchased from Harlan Sprague-Dawley Laboratories (Indianapolis, IN). For all surgical procedures, all efforts were made to minimize suffering to the mice. The University of Pittsburgh Institutional Review Board specifically approved this study.

### Tumor Xenograft Growth, Cell Culture and Serial Passage of TDECs

NCI-H460 human lung carcinoma (H460) cells were obtained from the American Type Culture Collection (Manassas, VA) and were cultured as previously described [16]. Authentication of H460 cells, as well as the serially-passaged H460-derived TDECs, was obtained by comparing the results obtained from the whole genome SNP array analysis with previously published results obtained by SKY analysis for NCI-H460 cells (see legend to Figures S2, S3, S4, S5, S6, and S7). Human umbilical vein endothelial cells (HUVECs) and EGM-2 EC-specific growth medium were purchased from Cambrex Bio Science (Walkerville, MD). Lentiviral packaging and infections to produce GFP-tagged and G418-resistant H460 cells were performed as previously described [16]. Initially, tumor xenografts were propagated by inoculating tagged H460 cells (10^6^) subcutaneously into the flanks of nude mice. The tumors were allowed to grow to the maximal allowable diameter of ca. 2 cm (typically 4–6 wks) and were then excised, minced, collagenase-digested, and passed successively through 100 µm and 40 µm mesh filters to obtain single cell suspensions (BD Falcon Cell Strainer, BD Biosciences, Franklin Lakes, NJ). Total murine and human tumor-associated endothelial cells (TAECs) were isolated from the bulk tumor cell population using two rounds of selection with magnetic beads coupled to human- and murine-specific anti-CD31 monoclonal antibodies (mAbs) [16]. The following day, cultures were examined using brightfield and ultraviolet microscopy to determine the percentage of GFP+ ECs (i.e. TDECs) and then subsequently selected for at least 7 days in G418-containing medium (500 µg/ml). The resulting human-derived cell population was termed “serial passage 0 (SP0) TDECs” and was routinely maintained in EGM-2 medium [16]. Serial passage was performed by re-inoculating 50,000 SP0 TDECs with 950,000 untagged H460 tumor cells (a 1∶20 ratio) into nude mice. Subsequently arising tumors were then used for repeat rounds of TDEC isolation and the generation of additional serially-passaged TDECs up to SP7. “Early passage” TDECs were generally comprised of SP1 cells and occasionally of SP0 cells, whereas “late passage” TDECs were comprised of SP6 or SP7 cells and occasionally of SP5 cells. Separate fragments of tumors were used for the preparation of frozen and paraffin-embedded sections.

### Evaluation of EC-specific Markers and Functions

Following their expansion, those TDECs not being used for serial passage studies *in vivo* were used to assess EC marker expression. For flow cytometry, at least 10,000 cells from short-term cultures of in vitro-selected TDECs were stained with anti-human CD31 and analyzed with a FACSCalibur flow cytometer (BD Biosciences). Data were analyzed and processed using BD FACSDiva software. For procedures requiring fixation, TDECs were grown on glass coverslips, fixed in PBS-4% paraformaldehyde and stained with antibodies against either human vWF or human ESAM as previously described [16]. We also quantified the uptake of rhodamine-tagged *Ulex europeus* lectin (E-lectin: Vector Laboratories, Burlingame, CA) and AlexaFluor 594-tagged acetylated low-density lipoprotein (AcLDL) (Invitrogen Molecular Probes) as previously described [16]. All photographs for fluorescence microscopy were taken with an Olympus Fluoview 1000 confocal microscope at a magnification of 40X–60X, except rhodamine-labeled E-lectin staining which was photographed at 20X. Adobe Photoshop CS2 (version 9.0, Adobe Systems, San Jose, CA) was employed for image analysis. qRT-PCR analysis of EC markers was performed as previously described [16].

### Tube Formation Assay

Characterization of tube formation in Matrigel was performed at 37°C as previously described [16] under normoxic or hypoxic conditions using a Hypoxic Glove Box incubator (Coy Laboratory Products Inc., Grass Lake, MI). Wells from 24-well plates were initially coated with Cultrex BME Growth Factor Reduced PathClear matrix (R&D Systems, Minneapolis, MN) (100 µl). TDECs, the original tumor cells (1000 cells/well) and HUVECs (10,000 cells/well) were then seeded in EGM-2 medium for the times and under the conditions indicated in the figures. Tube formation was typically assessed at 2, 6, and 10 d, at which points tubes were quantified and photographed using a Zeiss Axiovert 135 inverted microscope equipped with a Sony DXC-970MD 3CCD Color Video Camera. In assessing and quantifying TDEC tube formation, a “tube” was arbitrarily defined as the formation of an enclosed space surrounded by at least 10 cells linked end-to-end with a 1–2 cell layer thickness for at least 75% of the circle.

### Evaluation of Tumor Blood Vessels

Hematoxylin and eosin staining of paraffin-embedded sections from tumor xenografts was performed by the University of Pittsburgh Histology Core Laboratory. Criteria for tumor blood vessel identification were the presence of an identifiable lumen containing red blood cells.

### Whole Genome SNP Array, Chromosome Analysis, and qRT-PCR

SNP analyses were performed at The University of Pittsburgh Genomics and Proteomics Core Facility with using Ilumina 1 M DNA Analysis Bead Chips, which contains approximately 10^6^ SNPs with a mean genome spacing of 2.7 kb (median 1.7 kb). Analysis of the raw data was carried out using GenomeStudio software (version v2009.2). Changes in Log R Ratio and B Allele Frequency were used to identify gains or deletions within individual chromosomes. The software also allowed for identification of genes within the immediate proximity of duplicated or deleted genomic loci. The boundaries of the chromosomal alterations were determined using the identified SNP coordinates with the Ensembl Genome Browser 57 database. For qRT-PCR analysis of transcripts of genes identified by whole genome SNP array, total RNA was first purified using RNeasy mini-columns (Qiagen, Inc., Valencia, CA) (16) and 50 ng of input RNA was used in a SYBR Green-based assay (Quantitect, Qiagen) according to the directions of the supplier. Primers used to assess transcript levels of genes putatively affected by genomic alterations in late passage TDECs were selected from the MGH/Harvard PrimerBank site (http://pga.mgh.harvard.edu/primerbank) or were self-designed, with the exception of those for *NLGN1* [55]. These latter primers are described in Table S1 and were synthesized by International DNA Technologies (Coralville, IA). Confirmation of the predicted PCR product sizes was achieved by agarose gel electrophoresis and is shown in Figure S9. Data analysis was performed on an Applied Biosystems 7300 instrument (ABI, Foster City, CA). Relative quantification of gene expression data was carried out using a comparative *C*_T_ method and *GAPDH* was used as an endogenous control. Fold differences in the SP1 and SP7 mRNA levels relative to H460 RNA were then calculated by 2^−[Δ*C*T(SP1/7)−^ ^Δ*C*T(H460)]^.

### Statistical Analysis

Data were analyzed using either the one-tailed or two-tailed Student’s *t* test as appropriate.

## Supporting Information

Figure S1 **H460 tumor cells and early and late TDECs remain rounded after 2 d on Matrigel in normoxia.**
(TIF)

Figure S2 **SNP array profile analyses demonstrating genomic alterations for chromosome 2.** All other genomic alterations observed for late-passage TDECs are shown in Supplemental Figures S3–S7. In each case, the results shown (either Log R Ratio or B Allele Frequency) represent those that most clearly depict the chromosomal alteration, although both types of analyses were used for interpretations. It is noteworthy that for all chromosomes of H460-derived TDEC cell lines, a good agreement was found between the overall copy number based on B-allele frequency and log R ratio parameters and the number of chromosomes detected by SKY analysis in the H460 cell line previously reported [Liu et al. (2004) Modeling of lung cancer by an orthotopically growing H460SM variant cell line reveals novel candidate genes for systemic metastasis. *Oncogene* 23:6316-6324]. Chromosomal gains (green) and deletions (red) in late-passage TDECs relative to early passage TDECs are indicated as well as the base positions of the alterations on the chromosomes. The expanded regions below the SNP array plots show the genes within and adjacent to the sites of chromosomal alteration. The boundaries of the chromosomal alterations were determined using GenomeStudio software (version v2009.2) to identify SNP coordinates and the Ensembl Genome Browser 57 database.
(TIF)

Figure S3 **SNP array profile analyses demonstrating genomic alterations for chromosome 3.** A more detailed description of the SNP array analysis is given in the legend to Supplementary Figure S2.
(TIF)

Figure S4 **SNP array profile analyses demonstrating genomic alterations for chromosomes 4 and 5.** A more detailed description of the SNP array analysis is given in the legend to Supplementary Figure S2.
(TIF)

Figure S5 **SNP array profile analyses demonstrating genomic alterations for chromosomes 5 and 9. A more detailed description of the SNP array analysis is given in the legend to Supplementary Figure S2.**
(TIF)

Figure S6 **SNP array profile analyses demonstrating genomic alterations for chromosomes 13 and 14.** A more detailed description of the SNP array analysis is given in the legend to Supplementary Figure S2.
(TIF)

Figure S7 **SNP array profile analyses demonstrating genomic alterations for chromosome 20.** A more detailed description of the SNP array analysis is given in the legend to Supplementary Figure S2.
(TIF)

Figure S8 **qRT-PCR analysis of genes within or nearby chromosomal alterations detected in late passage TDECs by SNP analysis.** The relative amount of transcripts was quantified by a comparative CT method with *GAPDH* used as an endogenous control and the Δ*C*T values calculated for each sample. Results depicted in the graph represent mean values obtained (±SEM) from 3 independent sets of biological samples (i.e., 3 independent RNA preparations each of H460, SP1, and SP7 TDECs), except for *NRXN1*, *RAB3C*, and *ERCC8*, which represent results from two independent sets of RNA samples. *, *p*<0.05; **, *p*<0.01.
(TIF)

Figure S9 **Evaluation of qRT-PCR products following amplification from H460 RNA.** Products were electrophoresed on a 4% NuSieve agarose gel (Cambrex Bio Science Rockland, Inc., Rockland, ME).
(TIF)

Table S1 **qRT-PCR primers used in the analysis of Fig. S8.**
(TIF)
