# Supplementary figures and images for: In Vivo Evolution of Tumor-Derived Endothelial Cells
Source: PLoS One. 2012 May 18;7(5):e37138. doi: 10.1371/journal.pone.0037138 (PMC3356387; doi:10.1371/journal.pone.0037138)

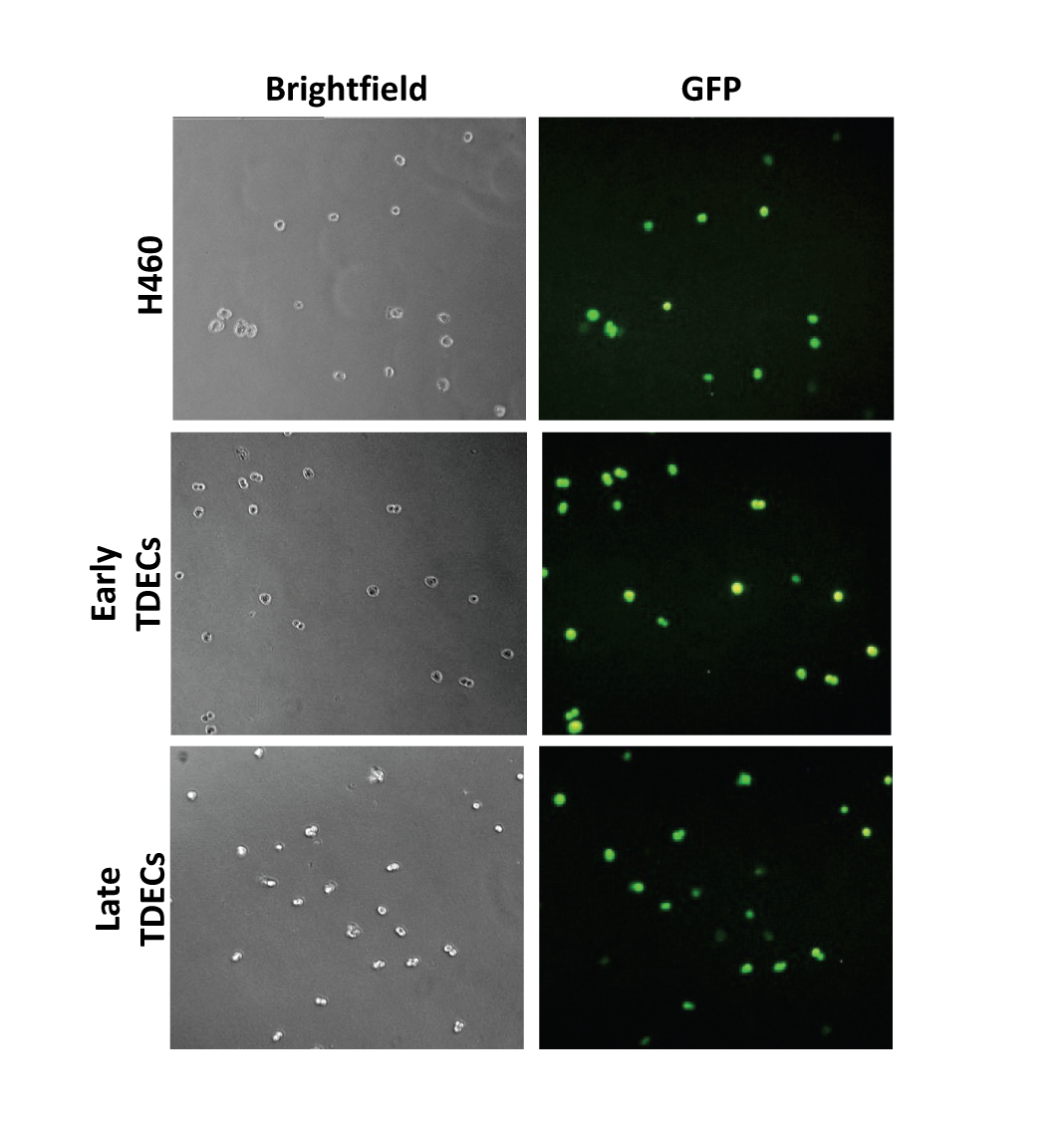

Supplement: Figure S1 — H460 tumor cells and early and late TDECs remain rounded after 2 d on Matrigel in normoxia. (TIF) [file pone.0037138.s001.tif]

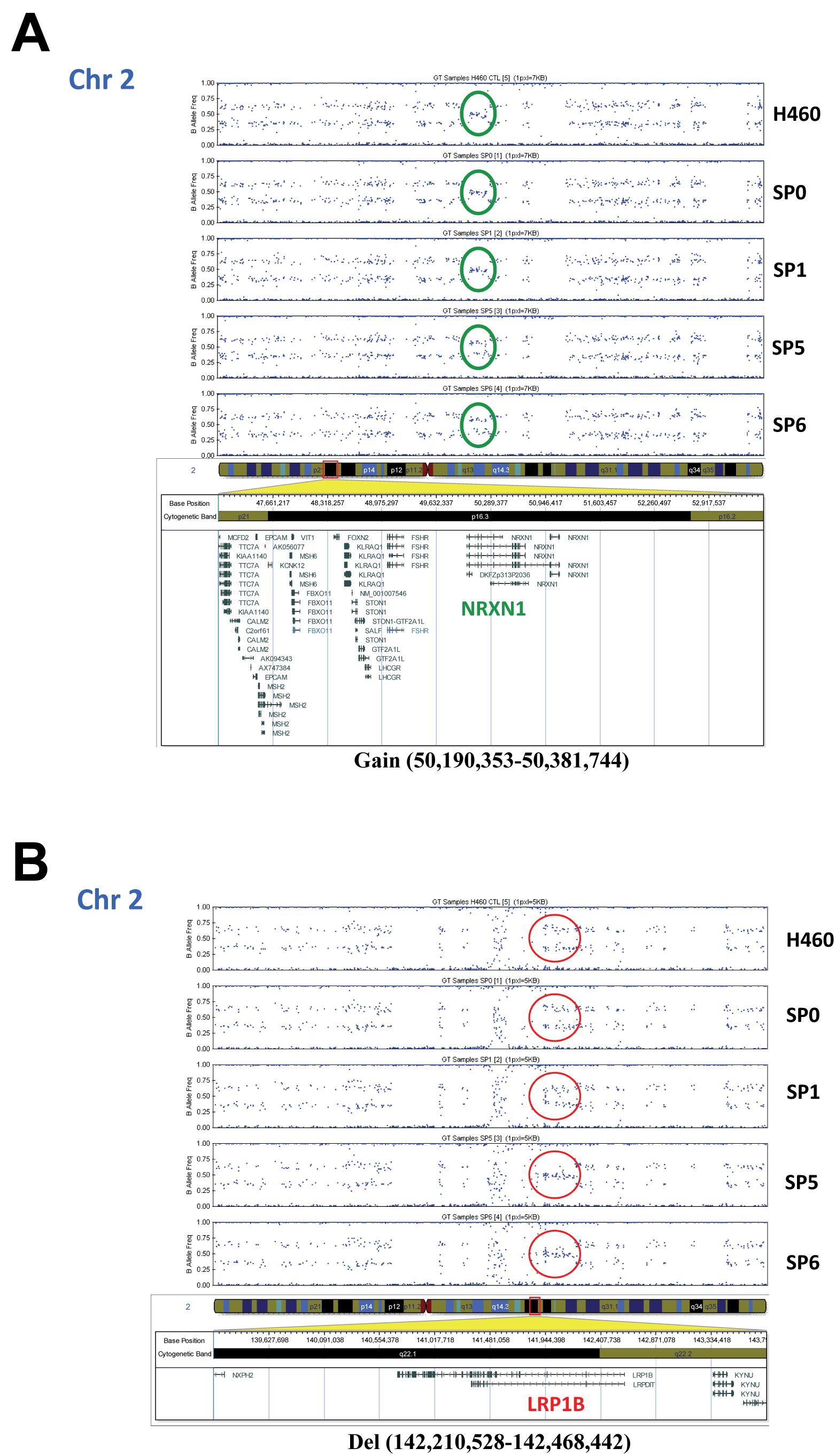

Supplement: Figure S2 — SNP array profile analyses demonstrating genomic alterations for chromosome 2. All other genomic alterations observed for late-passage TDECs are shown in Supplemental Figures S3–S7. In each case, the results shown (either Log R Ratio or B Allele Frequency) represent those that most clearly depict the chromosomal alteration, although both types of analyses were used for interpretations. It is noteworthy that for all chromosomes of H460-derived TDEC cell lines, a good agreement was found between the overall copy number based on B-allele frequency and log R ratio parameters and the number of chromosomes detected by SKY analysis in the H460 cell line previously reported [Liu et al. (2004) Modeling of lung cancer by an orthotopically growing H460SM variant cell line reveals novel candidate genes for systemic metastasis. Oncogene 23:6316-6324]. Chromosomal gains (green) and deletions (red) in late-passage TDECs relative to early passage TDECs are indicated as well as the base positions of the alterations on the chromosomes. The expanded regions below the SNP array plots show the genes within and adjacent to the sites of chromosomal alteration. The boundaries of the chromosomal alterations were determined using GenomeStudio software (version v2009.2) to identify SNP coordinates and the Ensembl Genome Browser 57 database. (TIF) [file pone.0037138.s002.tif]

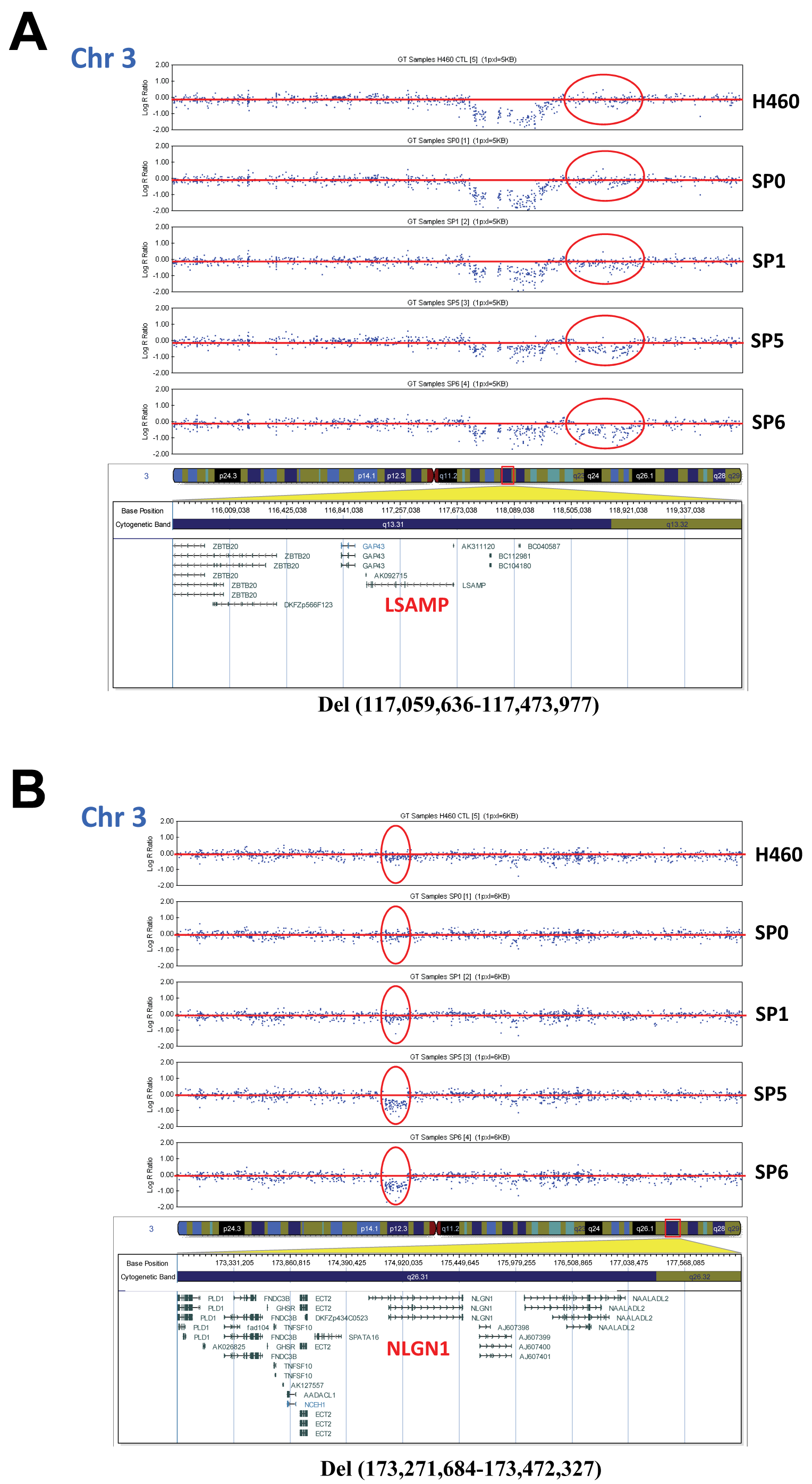

Supplement: Figure S3 — SNP array profile analyses demonstrating genomic alterations for chromosome 3. A more detailed description of the SNP array analysis is given in the legend to Supplementary Figure S2. (TIF) [file pone.0037138.s003.tif]

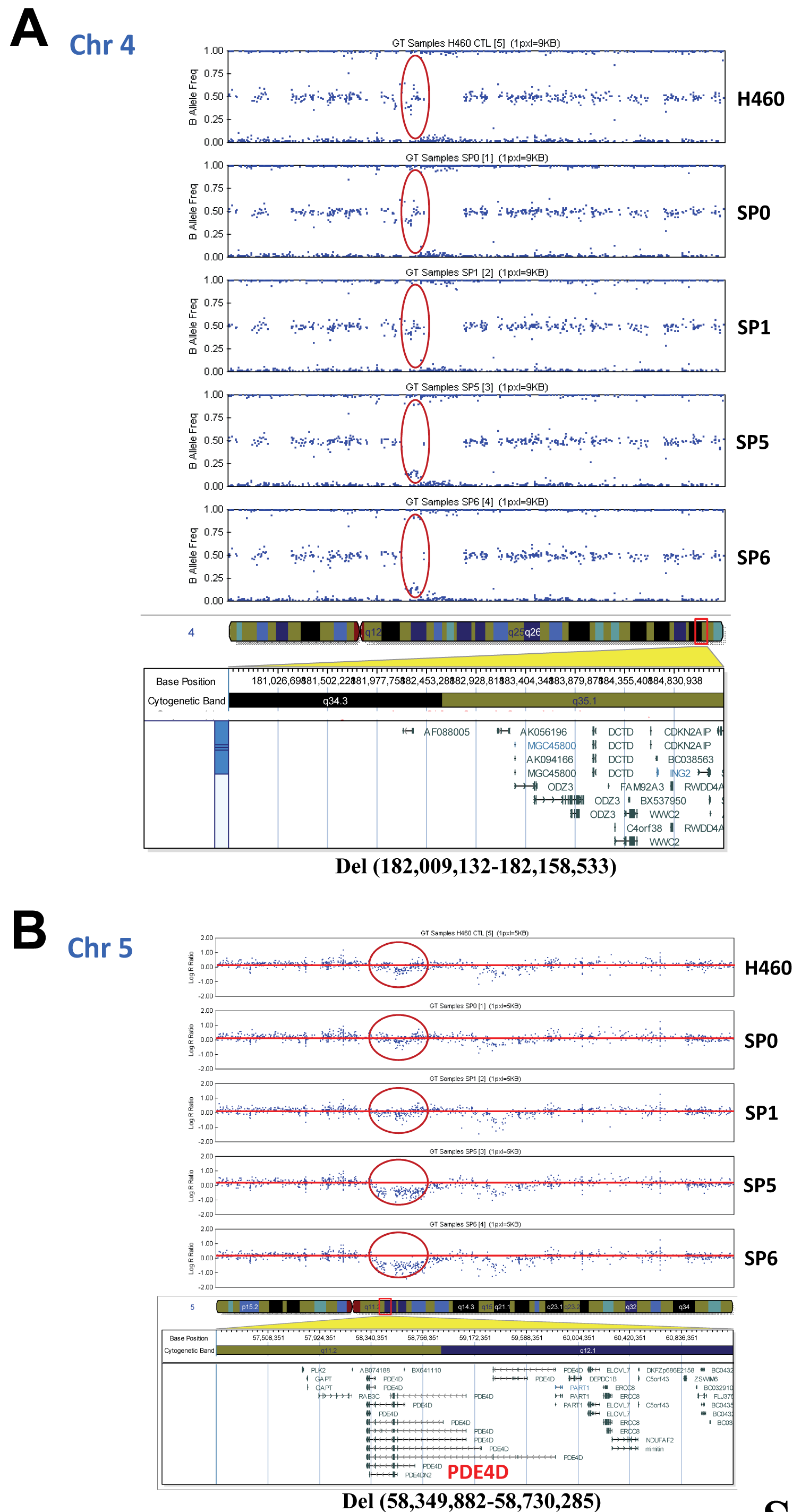

Supplement: Figure S4 — SNP array profile analyses demonstrating genomic alterations for chromosomes 4 and 5. A more detailed description of the SNP array analysis is given in the legend to Supplementary Figure S2. (TIF) [file pone.0037138.s004.tif]

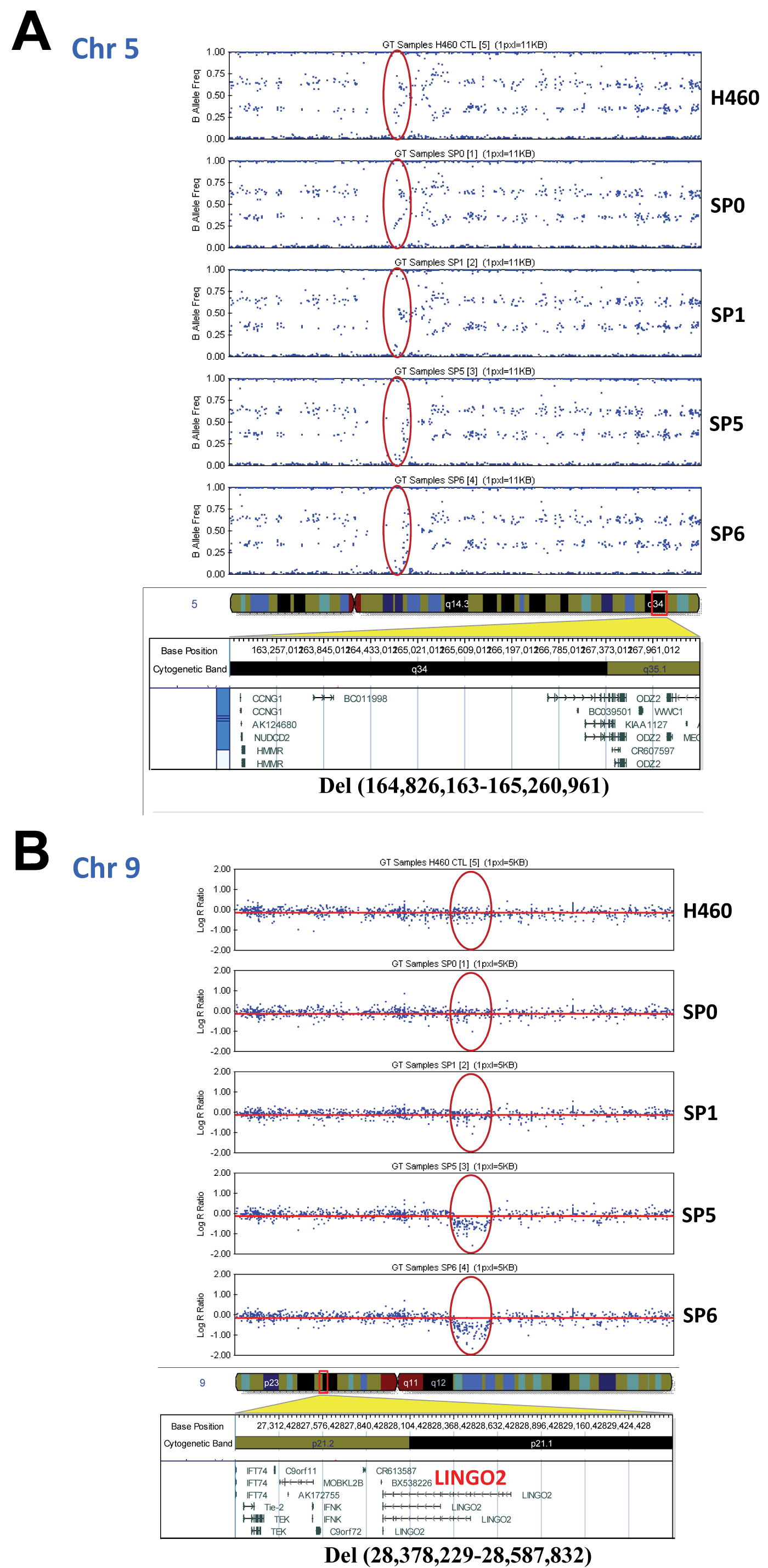

Supplement: Figure S5 — SNP array profile analyses demonstrating genomic alterations for chromosomes 5 and 9. A more detailed description of the SNP array analysis is given in the legend to Supplementary Figure S2. (TIF) [file pone.0037138.s005.tif]

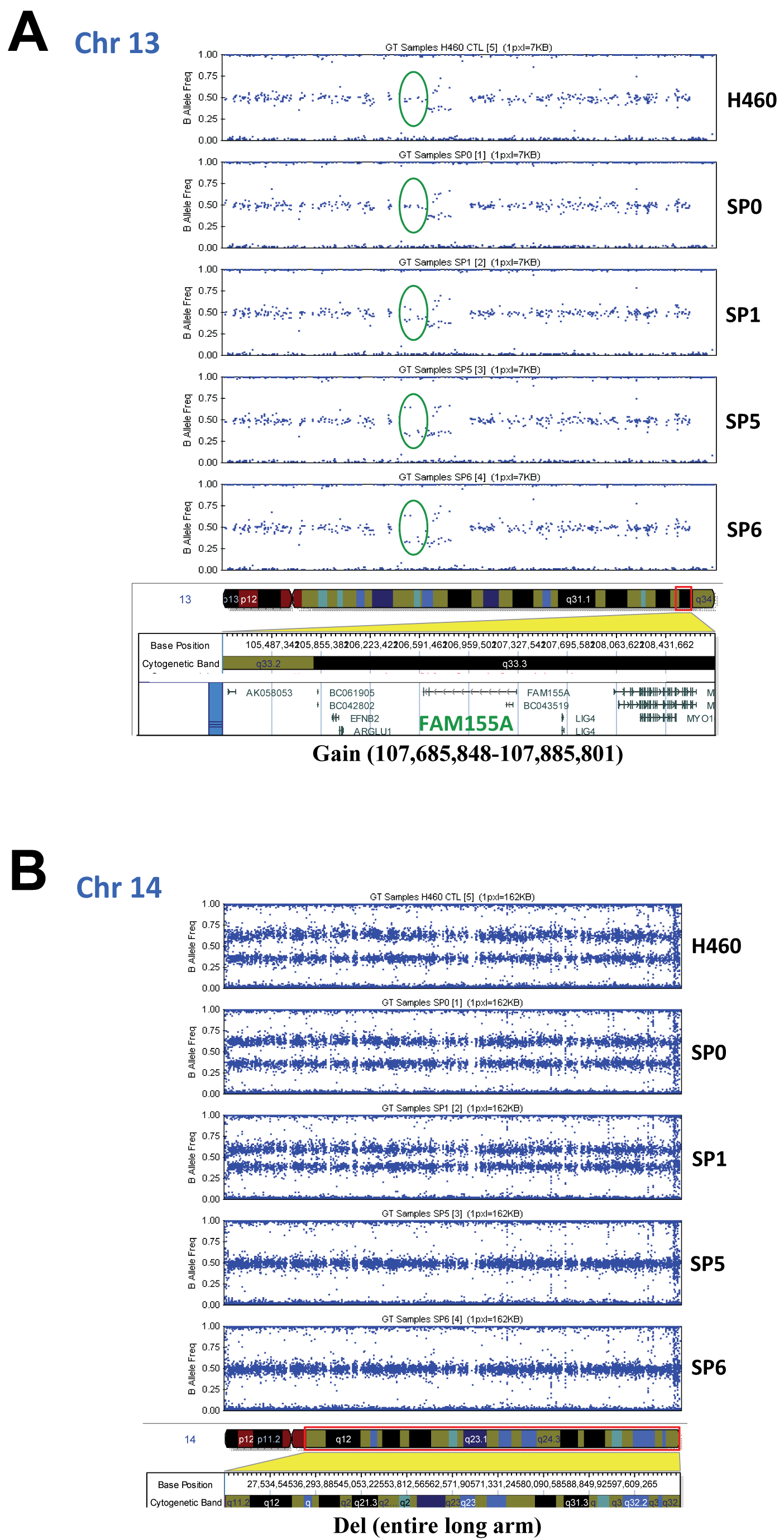

Supplement: Figure S6 — SNP array profile analyses demonstrating genomic alterations for chromosomes 13 and 14. A more detailed description of the SNP array analysis is given in the legend to Supplementary Figure S2. (TIF) [file pone.0037138.s006.tif]

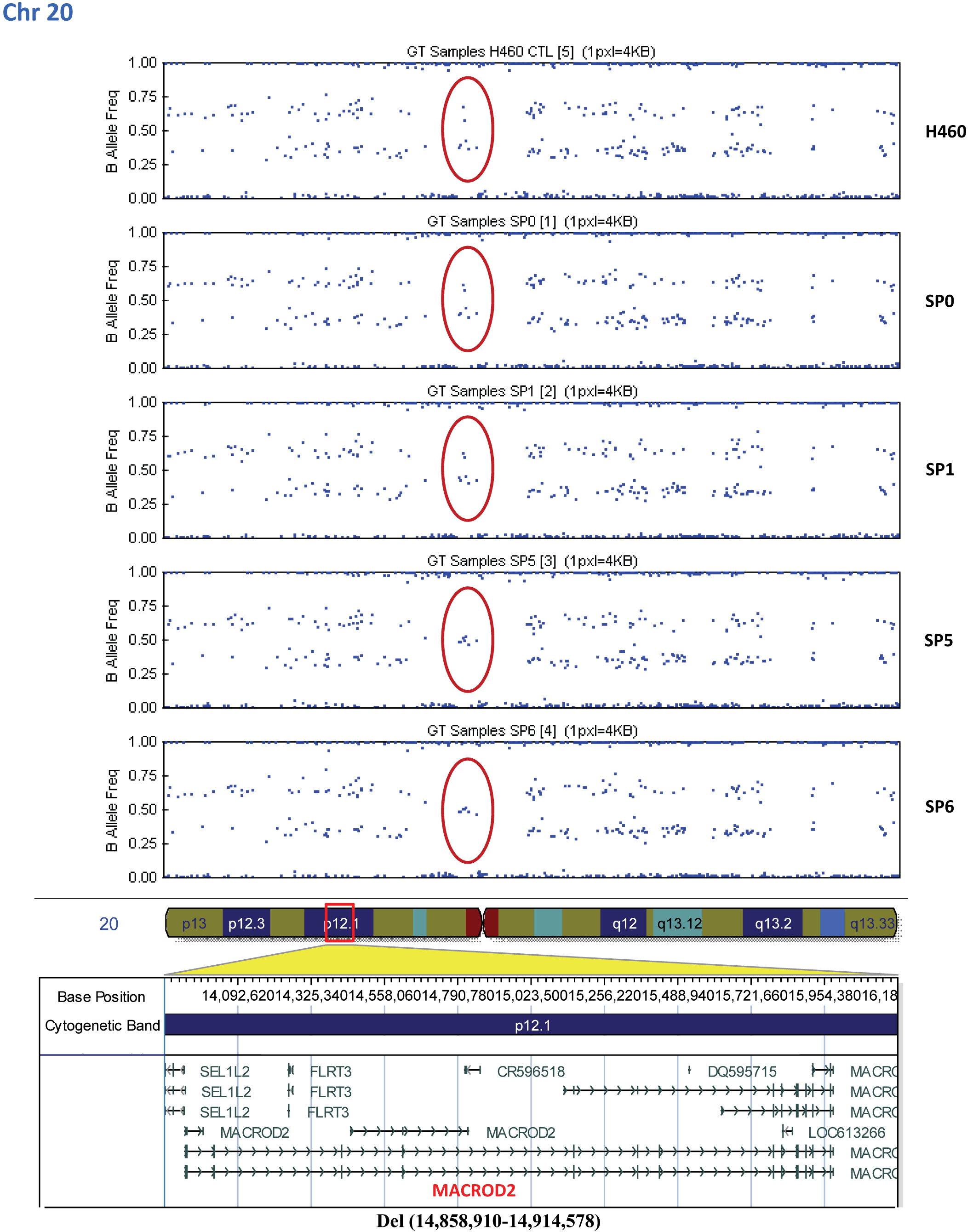

Supplement: Figure S7 — SNP array profile analyses demonstrating genomic alterations for chromosome 20. A more detailed description of the SNP array analysis is given in the legend to Supplementary Figure S2. (TIF) [file pone.0037138.s007.tif]

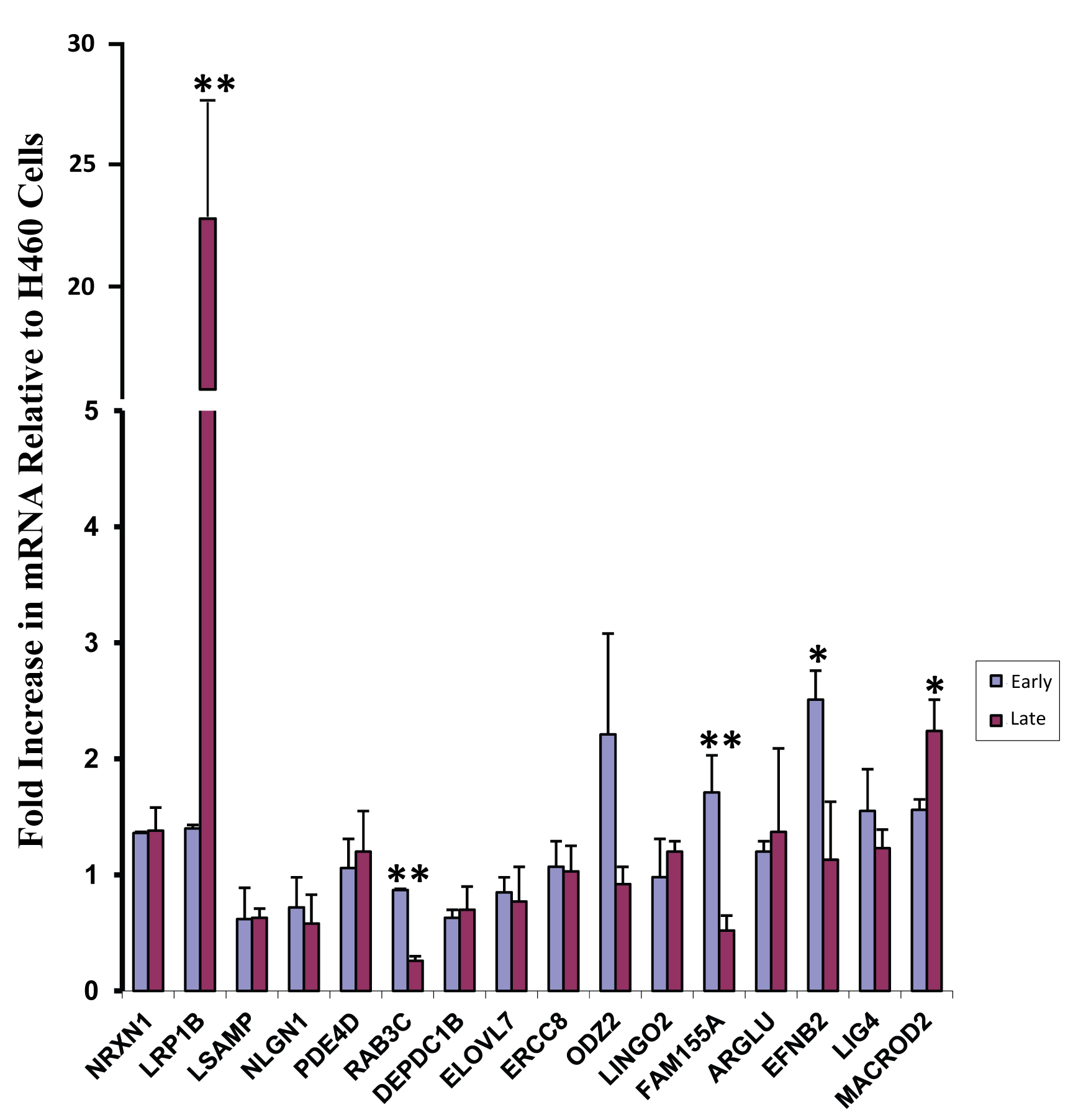

Supplement: Figure S8 — qRT-PCR analysis of genes within or nearby chromosomal alterations detected in late passage TDECs by SNP analysis. The relative amount of transcripts was quantified by a comparative CT method with GAPDH used as an endogenous control and the ΔCT values calculated for each sample. Results depicted in the graph represent mean values obtained (±SEM) from 3 independent sets of biological samples (i.e., 3 independent RNA preparations each of H460, SP1, and SP7 TDECs), except for NRXN1, RAB3C, and ERCC8, which represent results from two independent sets of RNA samples. *, p<0.05; **, p<0.01. (TIF) [file pone.0037138.s008.tif]

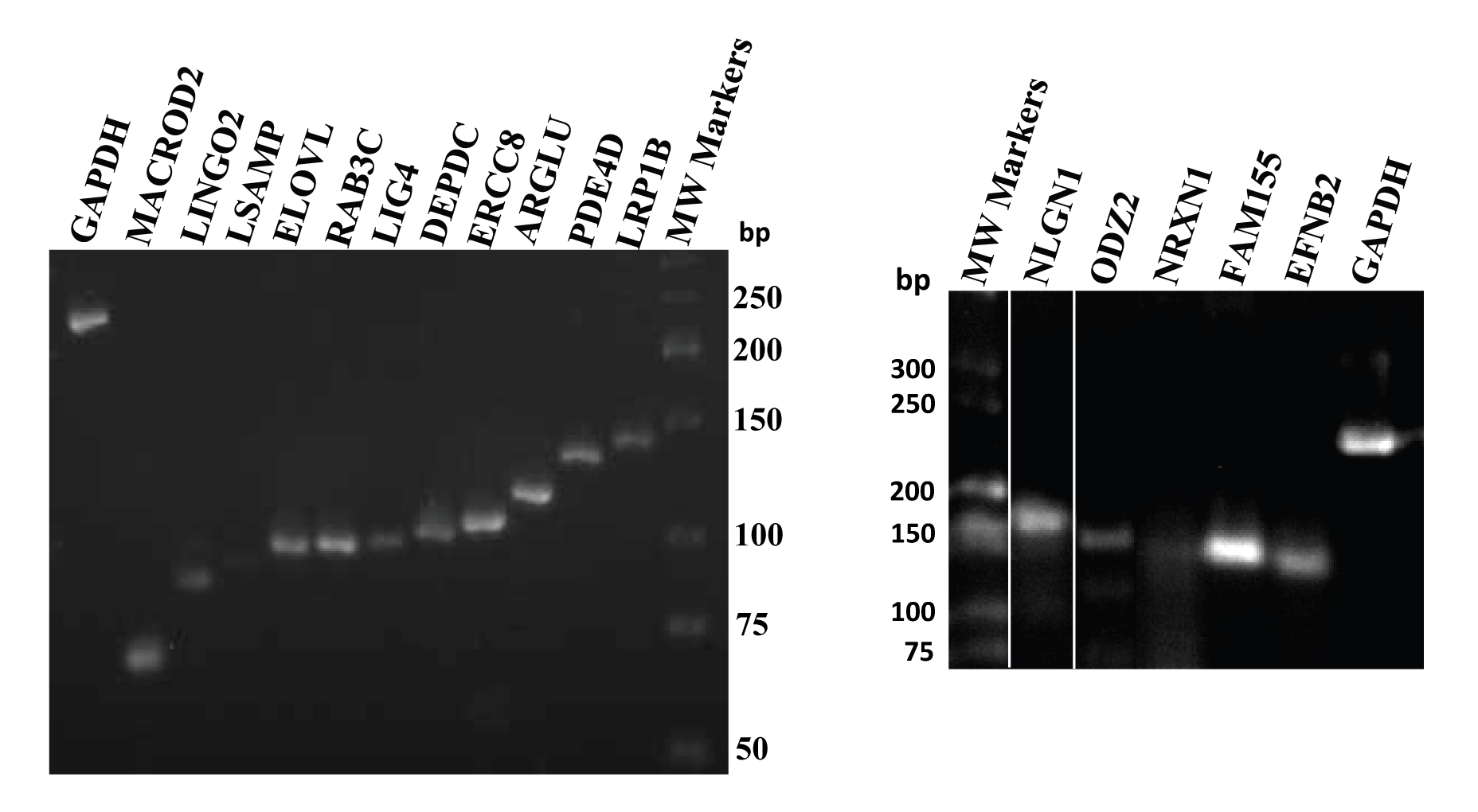

Supplement: Figure S9 — Evaluation of qRT-PCR products following amplification from H460 RNA. Products were electrophoresed on a 4% NuSieve agarose gel (Cambrex Bio Science Rockland, Inc., Rockland, ME). (TIF) [file pone.0037138.s009.tif]

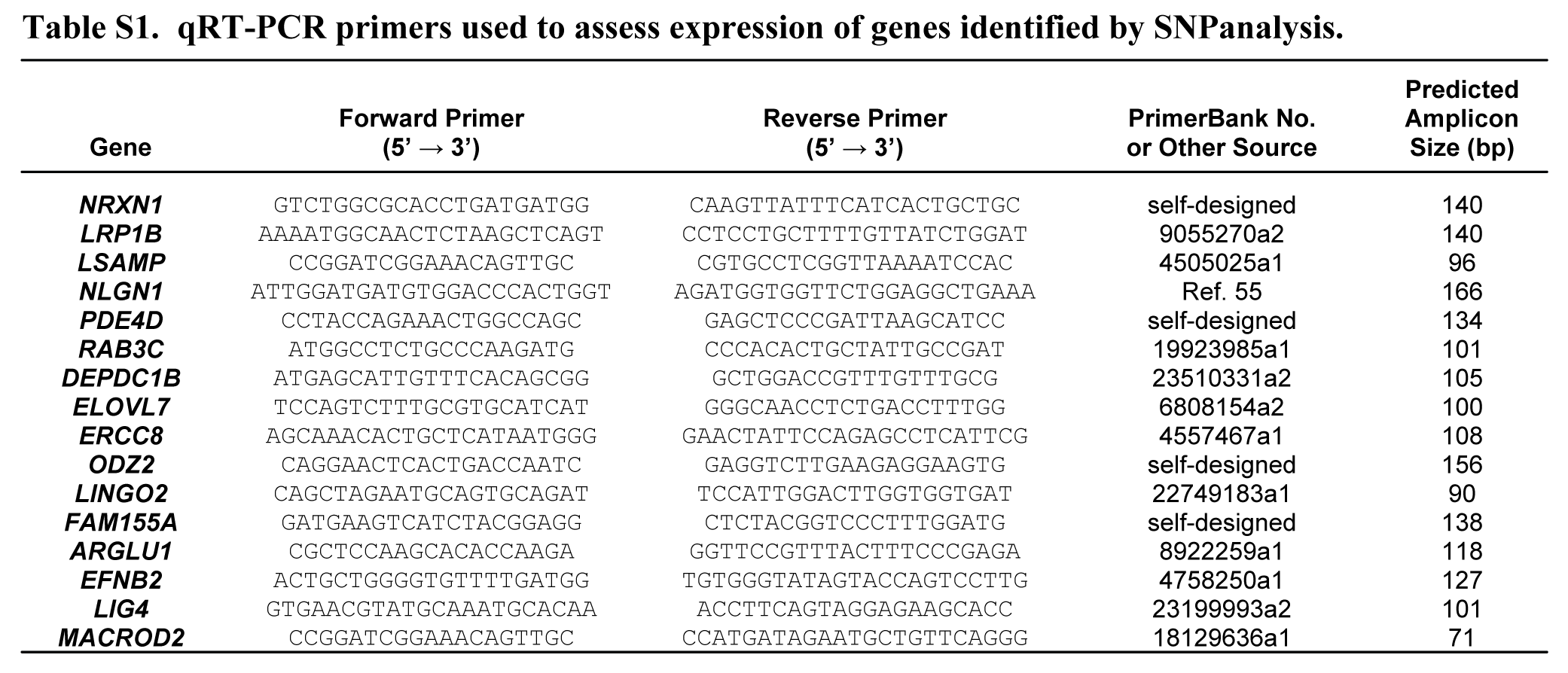

Supplement: Table S1 — qRT-PCR primers used in the analysis of Fig. S8. (TIF) [file pone.0037138.s010.tif]
